# Systemic BCG-Osis as a Rare Side Effect of Intravesical BCG Treatment for Superficial Bladder Cancer

**DOI:** 10.1155/2013/821526

**Published:** 2013-06-17

**Authors:** S. Lukacs, B. Tschobotko, N. A. Szabo, Andrew Symes

**Affiliations:** ^1^St Mary's Hospital Imperial College Healthcare NHS Trust, London Praed Street, City of Westminster, London W2 1NY, UK; ^2^King's College University Hospital NHS Trust, Denmark Hill, London SE5 9RS, UK; ^3^Brighton and Sussex University Hospitals NHS, Trust Eastern Road, Brighton BN2 5BE, UK

## Abstract

Intravesical Bacilli Calmette-Guérin (BCG) immunotherapy is a commonly used treatment for superficial bladder cancer. Although the treatment is well tolerated in 95% of cases, life-threatening side effects including BCG sepsis can occur. This report describes the case of an 82-year-old man with a background of lung disease. He developed septic shock and type two respiratory failure after receiving the sixth installation of intravesical BCG (TICE strain) immunotherapy for recurrent bladder Transitional Cell Carcinoma in situ. Despite the early initiation of broad spectrum antibiotics (tazocin and gentamicin), he remained pyrexial. There was a rapid deterioration, and on the second day of his admission, he developed type two respiratory failure secondary to Acute Respiratory Distress Syndrome (ARDS) prompting transfer to Intensive Care for Bilevel Positive Airway Pressure (BiPAP) Ventilation. The blood cultures taken before the induction of antibiotics results were negative. Increasing clinical suspicion of systemic BCG-osis prompted the initiation of antituberculosis therapy (ethambutol, isoniazid rifampicin) and steroids. Following six days of BiPAP and anti-tuberculosis therapy in ITU, his condition started to improve. Following a prolonged hospital stay he was discharged on long term ethambutol therapy. BCG-osis is a well-known though rare side effect of intravesical BCG therapy. We would like to highlight the importance of having a low threshold for starting anti-TB treatment.

## 1. Introduction

The term superficial bladder cancer refers to Ta, T1, and T carcinoma in situ (cis) lesions of any grade. Tumor grade and stage clearly have a strong influence on tumour progression and recurrence. The principal technique for the diagnosis and treatment of the superficial bladder lesions is endoscopy. Adjunctive intravesical therapy is available for residual disease or for tumour prophylaxis.

Since Morales et al. (1992) found an immunologically responsive tumour system, Bacillus Calmette-Guérin (BCG) has established itself as the most successful intravesical agent for the treatment and prophylaxis of different forms of superficial bladder cancer. The exact mechanism of BGC is still unknown. The bacilli attach to tumor cells through a novel fibronectin protein [1, 2]. Following attachment, the BCG is internalized into the tumour cell [3]. Raised levels of Interleukin-12 result in T helper (Th1) activation and increased interferon-*γ* production results in a positive ratio of CD4 helper and CD8 cytolytic T cells [4]. Furthermore, it has been suggested that intravesical BCG increases nitric oxide levels, which has been shown to inhibit tumour growth [5]. 

## 2. Case Report

We present the case of an 82-year-old man with a background of lung adenocarcinoma diagnosed in 1996 and treated with a left upper lobe lobectomy. Further he had Extrinsic Allergic Alveolitis (EAA) diagnosed in 2006. He also had multiple Squamous Cell Carcinoma lesions of the forearm, forehead and chest, as well as macular degeneration. Bladder Transitional Cell Carcinoma (TCC) was diagnosed in Zimbabwe in May 2009 with a histology of G2 pTa and was resected endoscopically. Surveillance flexible cystoscopies were unremarkable until 2011 when a positive bladder biopsy proved T carcinoma in situ (cis). BCG (TICE strain) induction therapy was initiated. The patient received one installation per week. The patient required a tetanus vaccination due to an unrelated incident, which indicated temporary suspension of treatment after the fourth installation. However during the first four installations, he suffered no side effects and the procedure was well tolerated. After a six-week pause, the BCG induction therapy continued with two further instillations.

**Figure 1:**
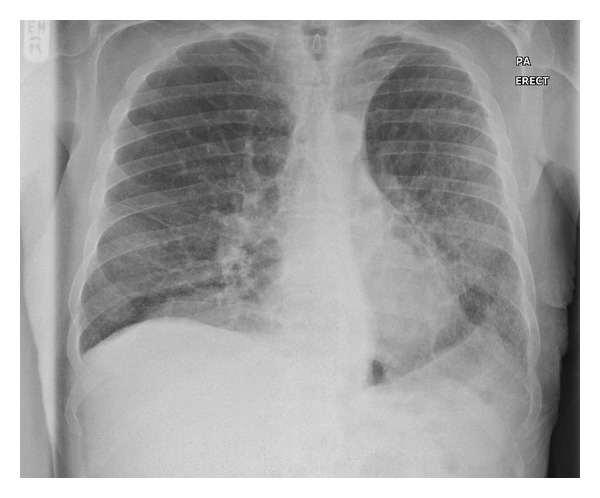
Chest X-ray on day one of admission.

Two weeks after the last BCG instillation, the patient was admitted with lethargy, loss of appetite, fever, and rigors. He had no respiratory or urinary symptoms. On examination, he was pyrexial (38.6°C), clinically dehydrated, hypotensive (85/34 mmHg), tachycardic (95/minute), and capillary refill was four seconds. ECG showed sinus rhythm. The cardiovascular and respiratory examinations were normal and the patient was saturating 96% oxygen on room air. Admission blood tests were unremarkable except for a mildly raised C-Reactive Protein (85 mg/L). Urine dipstick showed one plus of leukocyte and three plus of blood and ketones. A midstream urine culture and blood cultures were taken for aerobic bacteria, anaerobic bacteria, and tuberculosis (TB). Further samples were obtained for Influenza Polymerase Chain Reaction (PCR). Ultrasound of the urinary tract was normal. The chest X-ray (CXR) was unremarkable (Figure 1). Intravenous tazocin and gentamicin and fluids were started for suspected urological sepsis. 

**Figure 2:**
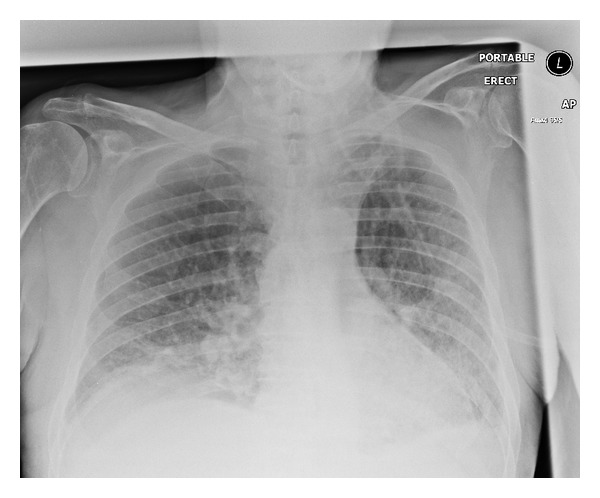
Chest X-ray on day two of admission.

Despite treatment, the patient suffered intermittent and worsening fever and developed type two respiratory failure prompting admission to the Intensive Care Unit (ITU) on day two of admission. A central venous catheter and arterial line were inserted, and Bilevel Positive Airway Pressure (BiPAP) was initiated. The CXR indicated that the type two respiratory failure was secondary to worsening Acute Respiratory Distress Syndrome (Figure 2). Respiratory physicians started antituberculosis triple therapy (ethambutol, isoniazid, and rifampicin) and prednisolone for suspected BGC-osis. Negative blood cultures and a strong clinical suspicion of BCG-osis prompted microbiology to advise discontinuation of tazocin and gentamicin. 

Overall, the patient spent six days on ITU. He required progressively lower ventilation pressures and was thus transferred to the respiratory ward. On the seventh day of anti-TB therapy, the patient showed deranged liver function tests and therefore isoniazid and rifampicin were discontinued. His liver function tests, normalized within two days of stopping these hepatotoxic agents. The patient required a forty-two-day hospital stay since he was significantly below his normal baseline function. The patient was discharged on long-term ethambutol and appropriate follow-up with the urology and respiratory teams. The manufacturer of the BCG product was notified of the adverse event. 

## 3. Discussion

BCG is a live attenuated strain of *Mycobacterium bovis*. Thus there is potential for serious illness and death. Acid Fast Bacteria (AFB) have been demonstrated in the bladder 16.5 months after completion of intravesical installations [6]. 

Most patients only experience mild side effects. These include fever and lower urinary tract symptoms including dysuria, frequency, and urgency that last for several days and worsen during the course of treatment [7]. Frank haematuria is also a common complication (40%) [7] and it is a relative contraindication for BCG delivery. 

More undesirable side effects are uncommon and include granulomatosis prostatitis, which can be an asymptomatic finding in 20% to 30% [8]. Symptomatic cases (1%) [8] require three months of anti-TB treatment [7]. Rarer complications of intravesical BCG immunotherapy include epididymitis (0.2%) [7] bladder contracture (<0.2%) [9], anaphylactoid purpura [10], vitiligo [11], and Reiter's syndrome with bilateral conjunctivitis and arthritis including the knees, ankles, and sacroiliac joints [12].

BCG-osis occurs following systemic absorption of BCG into the bloodstream. Risk factors include traumatic catheterization and a recent bladder tumour resection, where the integrity of the bladder mucosa is disturbed [13]. A fever exceeding 38.5°C lasting over 24 hours despite antipyretic therapy, or a recorded fever higher than 39.5°C, should prompt a high clinical suspicion of BCG-osis and should be treated empirically with Isoniazid (300 mg od) for three months [14]. It is generally more severe in patients who suffer from preexisting lung or liver disease. BCG sepsis is very rare [15], requiring resuscitation, ventilatory support, intensive care input, and triple anti-TB therapy. 

Infection with TB following intravesical BCG treatment has been categorized into “early” and “late” syndromes, presenting within or after three months, respectively [16]. The late presentations, such as in this case, are thought to result from “reactivation of infection after successful immunologic control of early dissemination” [16]. Infection has been reported as late as three years after the cessation of intra-vesical BCG treatment [16].

This case report indicates that although intravesical BCG immunotherapy is safe and usually produces only mild side effects [7], the adverse reactions can be severe and unpredictable. Therefore we should remain vigilant at all stages of treatment.
